# The RANK/RANKL/OPG system and tumor bone metastasis: Potential mechanisms and therapeutic strategies

**DOI:** 10.3389/fendo.2022.1063815

**Published:** 2022-12-16

**Authors:** Yan Zhang, Jingqi Liang, Peilong Liu, Qiong Wang, Liang Liu, Hongmou Zhao

**Affiliations:** Department of Foot and Ankle Surgery, Honghui Hospital of Xi’an Jiaotong University, Xi’an, China

**Keywords:** RANKL, osteoclast, bone homeostasis, signaling, cell

## Abstract

With the markedly increased diagnosis and incidence of cancer in the population, tumor bone metastasis has become a frequent event in tumor patients. Healthy bone integrity is maintained by a delicate balance between bone formation and bone resorption. Unfortunately, many tumors, such as prostate and breast, often metastasize to the bone, and the alterations to the bone homeostasis can particularly favor tumor homing and consequent osteolytic or osteoblastic lesions. Receptor activator of NF-κB ligand (RANKL), its receptor RANK, and osteoprotegerin (OPG) are involved in the regulation of the activation, differentiation, and survival of osteoclasts, which play critical roles in bone metastasis formation. High rates of osteoclastic bone resorption significantly increase fracture risk, cause severe bone pain, and contribute to homing tumor cells in bone and bone marrow. Consequently, suppression of the RANK/RANKL/OPG system and osteoclastic activity can not only ameliorate bone resorption but may also prevent tumor bone metastases. This review summarizes the important role of the RANK/RANKL/OPG system and osteoclasts in bone homeostasis and its effect on tumor bone metastasis and discusses therapeutic strategies based on RANKL inhibition.

## Introduction

Bones are the hardest organs in the human body, supporting the body’s weight and effectively maintaining sports function. Bone is also an endocrine organ, the maintenance of bone homeostasis is regulated by various factors in the internal and external environment, and the balance between bone resorption and bone formation plays an irreplaceable role in this process (1, 2). However, diseases of bone, such as cancer, trauma, and autoimmune diseases are increasing dramatically every year, leading to the pathological destruction of bone (3). All these pathological changes can bring a variety of health concerns, and bone metastasis is one of the most important complications of malignant tumors. A wide array of tumors, such as prostate and breast, often metastasize to the bone, and the alterations to the bone homeostasis can particularly favor tumor homing and consequent osteolytic or osteoblastic lesions (4, 5). When tumor cells are established in the bone microenvironment by disrupting bone homeostasis, it will result in increased bone resorption or bone formation. Subsequently, crosstalk between tumor cells and bone marrow cells disrupts bone homeostasis and promotes tumor growth. Therefore, more knowledge of bone homeostasis and how it affects tumor bone metastasis can support researchers in devising more effective strategies to prevent and treat tumor bone metastasis.

OPG/RANK/RANKL system is a critical signaling pathway regulating bone homeostasis, which includes receptor activator of nuclear factor-κB (RANK), receptor-activated nuclear factor-κB ligand (RANKL), and osteoprotegerin (OPG) (6). Multiple factors cause bone mass loss by activating the OPG/RANK/RANKL system, and overactivity of RANKL-RANK signaling can destroy bone homeostasis. The role of RANK/RANKL/OPG signaling in the regulation of bone homeostasis was first reported by Professor Boyle’s team in 1997 (7). This signaling plays a crucial role in mediating the balance between bone formation and resorption. RANKL is known as a central osteoclast (OC) differentiation factor. When RANKL occupies its receptor RANK, it activates intracellular signaling cascades in OC precursor cells to induce the formation of OC, and accelerates bone resorption by mature OC (8). OPG, also known as OC genesis inhibitor, as a competitive bait receptor of RANKL, reduces OC formation and activity by inhibiting the binding of the RANKL-RANK receptor (9).

Furthermore, the OPG/RANK/RANKL system has consistently been shown to be involved in tumor cell migration and development in bone and tumor bone metastasis (10, 11). Bone represents “an airport hub” of tumor cells, and the interactions in this microenvironment can determine the cell fate impacting the clinical outcomes of cancer. Finally, tumor cells and the bone microenvironment developed a vicious cycle of bone degeneration and tumor growth. In this study, we intend to summarize the significant function of the RANK/RANKL/OPG system in bone homeostasis and its effect on malignant bone metastasis.

### Overview of the RANK/RANKL/OPG system

**Figure 1:**
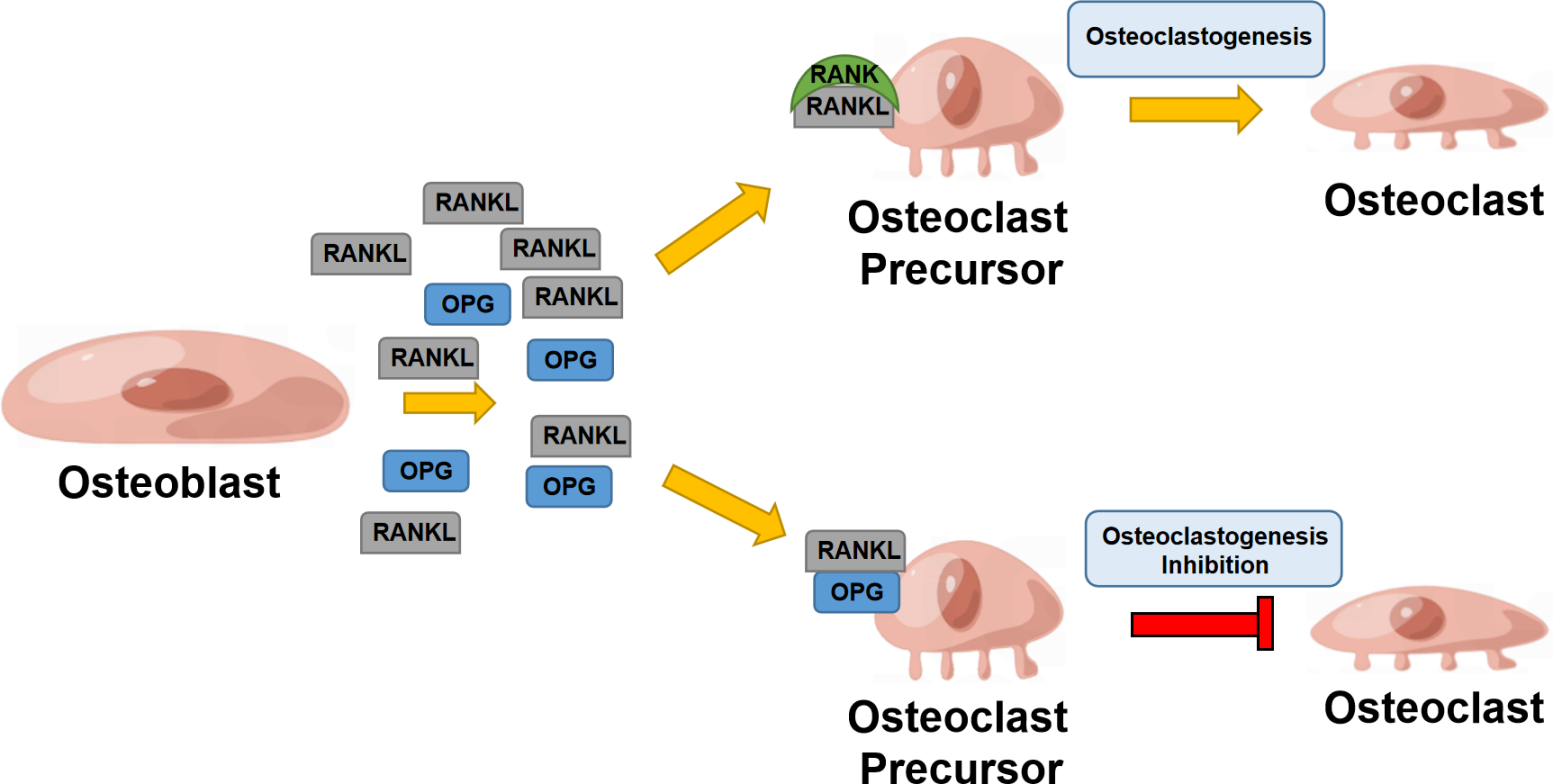
RANK/RANKL/OPG system in bone remodeling. Osteoblast secretes RANKL that binds to RANK, enhancing osteoclastogenesis; osteoblasts also secrete OPG, suppressing RANKL binding to RANK and inhibiting osteoclastogenesis.

RANKL is a crucial regulator of osteoclast differentiation and proliferation, mainly expressed by osteoblasts (OBs) and osteocytes in bone tissues (12, 13). In the RANKL/RANK/OPG system, RANKL can interact with RANK as its receptor and eventually induce osteoclast precursor maturation. OPG is known as a decoy receptor for RANKL, which can effectively inhibit RANKL-RANK interaction and mediate bone remodeling (14). RANKL is formed by osteoblasts and some other cells like activated T cells, in both cell associated and secreted forms, the latter derived by proteolytic action (15). RANK is capable of binding to RANKL and recruits TNF receptor-associated Factor 6 (TRAF6); TRAF6 can further activate NF-κB, mitogen-activated protein kinase (MAPK) family, tyrosine kinases c-Src and phosphatidylinositol 3-kinase (PI3K) signaling pathways (16). It was well-documented that RANK is enriched in a wide array of organs and functional cells, such as osteoclasts, vascular endothelial cells, mammary glands, and immune cells (17). It involves the regulation of bone remodeling, immune response, and mammary gland development (18) (**Figure 1**).

OPG, a 60 KDa glycoprotein that belongs to the TNF superfamily and is typically secreted by osteoblasts, has also been found in conjunction with lymphoid cell (19). OPG plays a predominant role in regulating bone homeostasis, and has seven structural domains, among which domains one to four support OPG to exert osteoclastogenesis function, which was considered the most important domain for bone remodeling (20). It was well-documented that inhibition of OPG can promote osteoclastogenesis and bone-resorbing activity (21). Interestingly, the regulatory roles of OPG in other tissues have attracted an accumulative research focus (22). Due to the existence of the heparin-binding domain which could bind glycosaminoglycans and proteoglycans (23), OPG mainly acts in local tissues. According to a recent study, local production of OPG, not circulating OPG, is essential for maintaining bone homeostasis. They concluded that OPG restricts its functions inside the tissue in which it is produced, highlighting the significance of local regulation of the RANKL system.

### RANK/RANKL/OPG system and bone homeostasis

Bone homeostasis is a complicated regulatory process mediated by multiple factors and the local microenvironment. Bone tissue is constantly remodeling, and OBs, OCs, and osteocytes are the main cell types involved in this remodeling process (24). During the bone remodeling process, the discrete zones of bone are degraded by OCs and replaced by the OBs-induced regenerated bone, allowing for bone repair and regeneration, and providing robust support for mechanical strengths. OBs are predominantly responsible for protein synthesis and matrix secretion to create new bones and maintain the integrity of skeletal tissues (25). Subsequently, the matured OBs can further differentiate into osteocytes which are the most abundant cells in bone tissue (26). The osteocytes form a lacunar-canalicular network connecting the neighboring osteocytes within the bone tissue and OBs and OCs on the bone surface (27). OCs act as bone orchestrators and initiate remodeling which is modulated by various local and circulating bioactive factors that contribute to bone homeostasis. Of note, osteocytes add “cement lines” in the remodeled bone matrix that define the reorganized osteons. This organization is supported by gap and desmosome junctions between osteoblasts and osteocytes providing intercellular communication and reinforcing separation from the system wide extracellular matrix (28). RANK/RANKL/OPG system is a classic signaling pathway regulating bone homeostasis in bone resorption and formation. Currently, accumulative interest was attracted to the critical role of the RANK/RANKL/OPG system in regulating OCs and OBs and the crosstalk between OBs and OCs during the bone remodeling process.

### Role of RANK/RANKL/OPG system in bone resorption

OCs are the principal cell type involved in bone resorption. It was widely demonstrated that multiple bioactive cytokines and factors derived from the OBs promote OCs differentiation and function (29). A wide array of bioactive cytokines and factors have been reported to involve in the regulation of osteoclastogenesis (30). During the past decades, knowledge of the molecular mechanisms that regulate OCs formation and differentiation has enriched increasingly since the discovery of the function of the RANKL/RANK/OPG system.

RANKL is commonly membrane-bound on osteoblastic cells or is secreted by activated T cells (31). Most of the stimulators of OCs formation can induce RANKL expression by OBs and osteoblastic stromal cells (32).

There has also been evidence of a soluble version of RANKL, which could result from either proteolytic processing or alternative mRNA splicing. In the ovariectomized mice model, the RANKL neutralising drug can markedly reduce osteoclastogenesis. Furthermore, the researchers found that the inhibition of RANKL-induced mitochondrial reactive oxygen species (ROS) production and the promotion of ROS-scavenging enzymes mainly contributed to the anti-osteoporotic effect (33). Noticeably, emerging evidence suggests RANKL is essential for OCs differentiation from their precursors and the survival and activation of mature OCs (34). Hodge et al. (35) reported that macrophage colony-stimulating factor (M-CSF) could enhance OCs survival and augment RANKL-induced activation of mature OCs.

RANKL exerts its effect *via* binding and activating RANK. In ovariectomized mice, the inactivation of RANKL can lead to an inhibition of OCs maturation and ameliorate ovariectomy-induced osteoporosis (36). Furthermore, the *in vitro* results indicated that the functional deficiency of OCs is consistent with the expression of RANK on the surface of OCs precursors (37). RANKL expression by osteoblastic stromal cells is modulated by bioactive cytokines and factors that stimulate bone resorption (38). Colony-stimulating factor-1 (CSF-1), tumor necrosis factor (TNF), prostaglandins, steroids, parathyroid hormone (PTH), PTHrP, and IL-1/6/11 are among the bioactive cytokines and factors that control osteoclast production and activity, and consequently, normal bone remodeling (39). In a prior study, professor Boyle’s group had generated RANK nullizygous mice to investigate the underlying mechanisms of the RANKL/RANK/OPG system in the regulation of bone resorption and remodeling processes (40). In this study, they found that the primary osteoclastogenic action of the bioactive cytokines is indirect to OCs and the activation of their regulatory function is dependent on the RANK/RANKL signaling. Taken together, the cellular communication between RANKL and RANK is the utmost positive signal for osteoclastogenesis which is partially counterbalanced by the activity of OPG.

### Regulation of osteoblastic activity by the RANK/RANKL/OPG system

OBs are involved in the modulation of bone remodeling by forming the bone matrix. Especially, osteoblasts are predominantly responsible for depositing calcium phosphate crystals like hydroxyapatite and producing components of the bone matrix like type I collagen (41). Subsequently, as the bone matrix enters the mineralization phase, osteoblasts contribute to the production of many proteins that are closely related to the mineralized matrix *in vivo*. Furthermore, OBs are responsible for osteoclastogenesis and the survival of mature OCs. OBs produce RANKL, which can induce OCs precursor differentiation into OCs by interacting with RANK. Subsequently, the formation of the RANK‐RANKL axis can further induce the trimerization of RANK and the activation of TRAF6. In addition, modulation of the RANKL/OPG ratio is an important way for OBs to mediate osteoclastogenesis (42). OBs synthesize OPG, which acts as the competitive receptor of RANK/RANKL binding, thereby inhibiting RANK activation and ameliorating the following osteoclast activation (43).

Although the effect of the RANK/RANKL/OPG system on bone resorption is well reported, its role in the regulation of OBs survival and osteoblastic differentiation remains elusive and attracts increasing research interest. Previous studies demonstrated that OPG is not capable of inducing bone formation and osteoblastic differentiation, and RANKL can affect OBs function, proliferation, and differentiation by other underlying mechanisms (44, 45). For instance, professor Aoki’s group (46) found that RANKL acts as a receptor of OBs to promote osteoblastic differentiation. Mechanistically, the *in vitro* results indicated that the induction of the clustering of RANKL molecules on the plasma membrane was the main mechanism of the promoted osteoblastic differentiation.

### The role of the RANK/RANKL/OPG system in tumor bone metastasis

A delicate balance between bone formation, mediated by osteoblasts, and bone resorption, mediated by osteoclasts, controls bone homeostasis. In tumor bone metastasis, the bone represents “an airport hub” of cancer cells deriving from different types of primary tumors. Malignant cells within the bone can reduce bone formation. RANKL can induce osteoclastogenesis and bone resorption and plays a crucial role in mediating bone remodeling through binding to RANK. Conversely, OPG blocks this process by interacting with RANKL and ameliorating bone disruption (47). The role of RANKL in developing breast and prostate tumors was well-documented in prior studies. In a pioneering study, Nolan et al. (48) found that RANK (+) progenitors are a crucial target population in breast cancer patients with BRCA mutation *via* the identification of two subsets of luminal progenitors in histologically normal tissues. In addition, they performed comprehensive assays to determine the regulatory role of RANK/RANKL in the development of breast cancer, indicating a crucial role for RANK/RANKL signaling in tumor initiation and providing a research direction and robust support for the anti-RANKL clinical studies, such as denosumab, in the treatment of breast cancer. Similarly, Sigl et al. (49) found that the inactivation of RANK in the mammary epithelium could delay onset, decrease incidence, and ameliorate the development of BRCA1 and p53 mutation-driven mammary cancer by using two kinds of conditional gene knock-out mice models. Specifically, inhibition of RANKL/RANK signaling was demonstrated to suppress proliferation, migration, and expansion of both murine BRCA1 and p53 mutant mammary stem cells and mammary progenitors. This study provided considerable evidence for RANK as a critical therapeutic target in breast cancer. The key role of RANK in the development of prostate tumors was also demonstrated in prior studies (50–52). In a recent study, Niu et al. (53) introduced a novel anti-RANKL strategy for ameliorating prostate tumor-derived bone metastasis. Specifically, they constructed biomineralized metal-organic framework nanoparticles carrying protein toxins with bone-seeking and CD44-receptor-targeting functions. The *in vivo* results indicated that the functional nanoparticles could exert synergistic intervention of the crosstalk between bone cells and tumor cells *via* combination with RANKL antibody. All these studies demonstrated the crucial roles of RANK/RANKL in tumor bone metastasis and supported that anti-RANKL therapy represents a promising strategy for ameliorating bone metastasis.

Furthermore, tumor cells within the bone can also release various bioactive cytokines to regulate the activity of the RANK/RANKL/OPG system and induce bone destruction (54). On the other hand, it is well-documented that RANKL is involved in the migration, invasion, and proliferation of malignant cells, and OPG increases the survival of tumor cells (55). Therefore, it is of great necessity to master more in-depth knowledge of the relevance between the RANK/RANKL/OPG system and tumor bone metastasis.

### The characteristics of bone metastasis

The molecular mechanism of tumor bone metastasis remains elusive. As the main cellular elements of bone tissues, OCs, OBs, and osteocytes are involved in the metastasis process. OCs are multinucleated cells derived from monocytes that can induce bone matrix degradation by providing an acidic environment and releasing a variety of protein enzymes (56). OBs are derived from pluripotent mesenchymal stem cells that promote bone matrix formation and regeneration (57). It was reported that a wide array of bioactive cytokines could be released from bone tissue, which is crucial for tumor growth and metastasis and was assumed to be one of the main reasons bone is a preferred distant organ for tumor metastasis. The host microenvironment and the local environment play a role in the tumor’s ability to successfully engraft from the primary site to bone (58).

Bone resorption mediated by OCs *via* protease action and the acidification of the local bone microenvironment is principally responsible for the osteolysis in tumor bone metastases (59). Chronic intractable pain is one of the most common symptoms of bone metastases, indicating that OCs play a predominant role in the bone metastasis process. On the one hand, tumor cells can release direct pain signals to induce metastatic cancer pain locally. On the other hand, bone resorption ought to deteriorate by tumor cells-induced osteoclastogenesis, which is assumed to be the principal mechanism (60). It is well-documented that many acid-sensing channels are found in the sensory neurons of the periosteum. OCs can release various enzymes to create a more acidic microenvironment, making the neurons more sensitive and inducing pain (61). This process of bone remodeling increases the risk of fractures and ultimately results in pathological fracture and spinal cord compression in patients with bone metastases. Given the crucial role of OCs in bone metastasis, studying bone homeostasis and how it relates to tumor bone metastasis will allow researchers to develop more effective strategies in orthopedic disease research.

### The role of the RANK/RANKL/OPG system in bone metastases

It is well-documented that RANK is mainly derived from OCs and their progenitors. Interestingly, emerging evidence indicates that RANK is also expressed on tumor cells and appears to be involved in the tumor metastasis process (62). The role of RANK and the RANK/RANKL/OPG system in bone homeostasis and tumor metastasis is important for uncovering the mechanism of tumor bone metastasis.

RANKL, RANK, and OPG are the main members of the TNF and TNF-receptor superfamily capable of promoting differentiation, activation, and survival of OCs. Stromal osteoblast cells support osteoclast differentiation by their ability to secrete IL-6 and RANKL in response to PTHrP, vitamin D3 and TNF-α (63). Moreover, the ratio of RANKL/OPG determines the bone resorption level.

**Figure 2:**
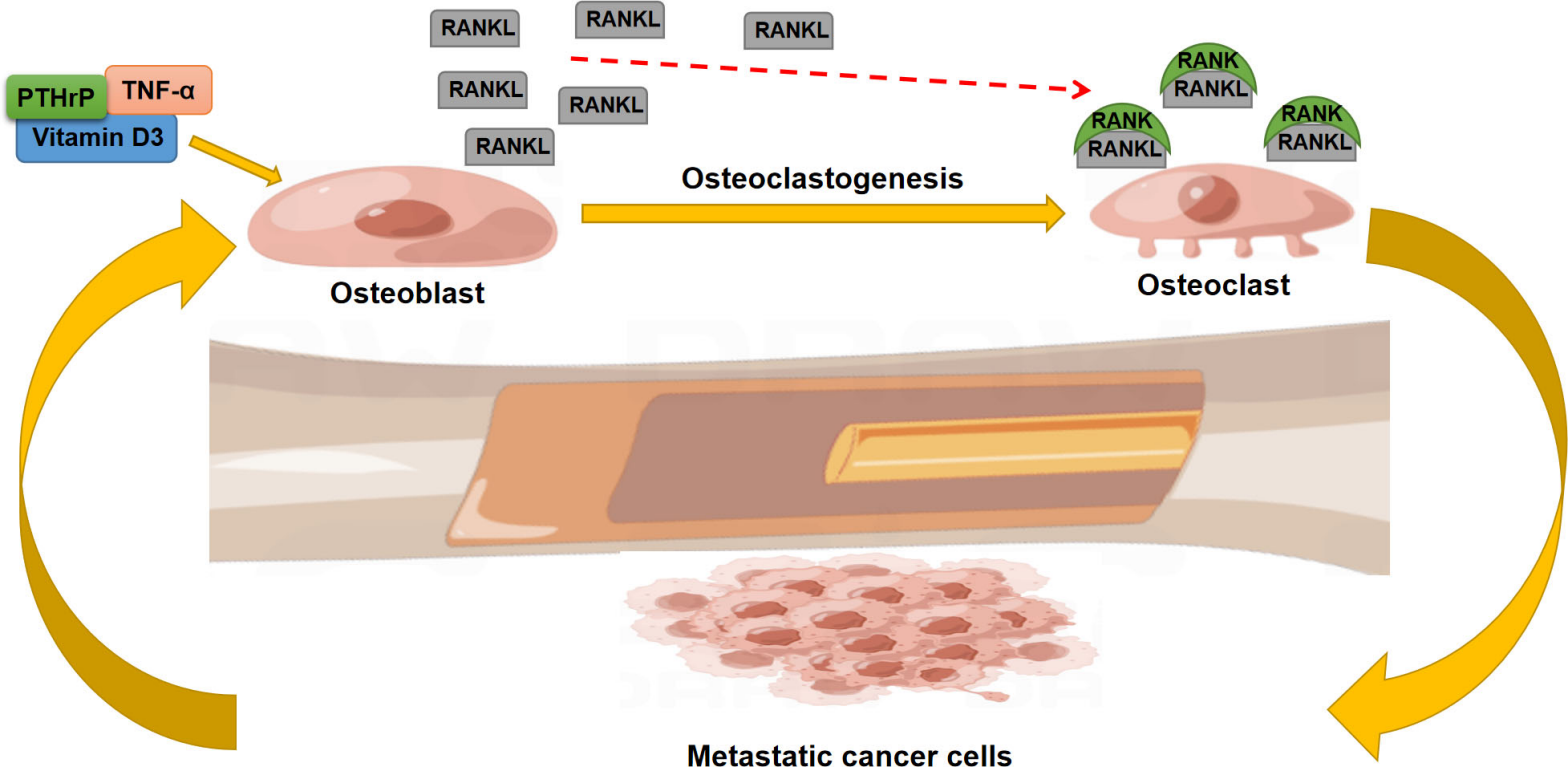
The role of the RANK/RANKL axis in the regulation of bone metastasis.

Owing to this regulatory effect, the RANK/RANKL/OPG system displays a predominant role in the development of bone metastases (64): RANKL activates osteoclast-mediated bone resorption with a consequent release of matrix growth factors. These factors can promote the growth of tumor cells and create a positive feedback mechanism (**Figure 2**). In addition, cytokines, chemokines, growth factors, and hormones derived from tumor cells enhance RANKL expression through PTHrP-induced OPG suppression (65). Ni et al. extracted the tumor-derived exosomal lncRNA-SOX2OT and found that this exosomal lncRNA can promote tumor bone metastasis by activating TGF-β/pTHrP/RANKL signaling (66). Moreover, the *in vivo* results indicated that inhibition of RANKL signaling could effectively ameliorate bone metastasis and osteoclastogenesis (67).

### Denosumab and therapeutic strategies

RANKL/RANK/OPG system is a key pathway for balancing osteogenesis and osteoclastogenesis. RANKL and RANK combine to activate osteoclastic activity and promote osteoclastogenesis. OPG is a decoy receptor for RANKL, which inhibits the RANKL/RANK pathway and promotes osteogenesis. The ratio of OPG/RANKL determines bone formation or resorption. Therefore, blocking the RANK/RANKL system may present novel therapeutic opportunities for managing skeletal disorders and tumor bone metastases.

Late-stage clinical trials with denosumab, a well-known RANKL inhibitor, are currently being conducted to treat osteoporosis and skeletal diseases brought on by tumor metastasis. Like OPG, denosumab binds to RANKL and prevents the development of the RANKL-RANK axis. This antibody does not interact with other molecules and binds RANKL alone (68). Many pre-clinical trials previously verified the therapeutic efficacy of denosumab, which showed that denosumab could significantly increase femoral and vertebral bone strength 12, 24, and 36 months after initial treatment (69). In addition, the bone matrix mineralization increased with denosumab treatment for up to 5 years to nearly physiologically normal levels and remained steady after that (70).

Owing to its robust function in suppressing mature osteoclast proliferation and osteoclastic differentiation, it was assumed that denosumab could suppress the osteoclast-tumor cell axis and ameliorate bone lesions induced by tumor metastasis. Lipton et al. compared denosumab and zoledronic acid to treat bone metastases from advanced breast cancer, showing that denosumab can markedly treat and delay bone metastases from breast cancer (71). Based on the clinical observation, they concluded that denosumab outperformed zoledronic acid in treating patients with advanced cancer who had bone metastases by reducing skeletal-related events with excellent safety and convenience. However, the efficacy of current anti-RANKL therapy is far from expected. Exploring the mechanisms of adverse reactions to denosumab could provide the direction for developing new RANKL inhibitors with more specificity and safety. Due to a greater understanding of the mechanisms underlying the onset of bone metastasis, more bone-targeting agents and drugs, such as the anti-RANKL drug denosumab, have been successfully developed over the past few decades. Cadieux et al. (72) performed a detailed summarization and analysis of the efficacy of denosumab in treating tumor bone metastases in the past ten years. It was concluded that inappropriate use and dosing of denosumab might lead to an unsatisfactory result for patients, and it is highly desired that more studies optimize the clinical administration of patients using denosumab with better safety and efficacy. There was a notable lack of benefit for bone metastasis-free survival in patients treated with adjuvant denosumab for breast cancer, highlighting the need for additional research into the potential role of denosumab in early-stage malignancies (73). More importantly, increasing concerns were shed on the denosumab discontinuation, which may bring many uncertainties (49, 52, 73, 74). Denosumab discontinuation was associated with a severe rebound effect consisting of a two-year increase in bone remodeling markers and a loss of the gained bone density; this is an issue with the period management of denosumab therapy. Bisphosphonate was considered a potential strategy at denosumab discontinuation, which may reduce the risk of bone loss. Noticeably, this strategy should be rigorously monitored, and proper adjustment of treatment should be given if bone remodeling is out of control (75).

### Challenges and perspectives

In-depth knowledge of the complicated mediation of the RANKL/RANK/OPG system in bone homeostasis and tumor bone metastasis is of great necessity to prevent tumor bone metastasis and related bone lesions. In this review, we focused on the multifunctional roles of the RANKL/RANK/OPG system. Although this system is the classic signaling pathway in the regulation of bone homeostasis, emerging evidence has widely demonstrated that it also plays an important role in tumor bone metastasis. Suppression of the RANKL/RANK/OPG system may therefore provide novel research direction for the treatment of skeletal diseases and tumor bone metastasis.

The idea that RANKL is a potentially significant target for bone metastases treatment is supported by the demonstrated therapeutic efficacy of RANKL inhibition in animal bone metastases models, which depict a variety of osteoblastic and osteolytic bone lesions. However, the therapeutic strategies targeting the RANKL/RANK/OPG system to stop bone resorption deterioration need more in-depth research regarding the interaction between bioactive cytokines such as interleukins, PTH, and growth factors with the activation or suppression of the RANKL/RANK/OPG system.

The cellular and molecular processes entailed in bone metastasis biology are still being further defined, and this will probably result in novel and better treatment approaches to treat bone metastasis. More studies should be done on the clinical significance of neutralizing RANKL in malignancy and skeletal diseases. Uncovering the molecular mechanisms of the RANKL/RANK/OPG system in bone tumor metastasis requires a deeper understanding of the roles played by each domain, particularly the RANKL-, TRAIL-, and heparin-binding domains.

## Author contributions

YZ and JL wrote the manuscript. PL, QW and LL reviewed some papers and participated in the preparation of the manuscript. HZ provided the idea and supervised this study. All authors contributed to the article and approved the submitted version.
